# Continuous Glucose Monitoring Intervention for Hispanic Adults With Type 1 Diabetes Receiving Care in a Federally Qualified Health Center: Protocol for a Mixed Methods, Pragmatic Pilot Randomized Controlled Trial

**DOI:** 10.2196/60583

**Published:** 2026-06-02

**Authors:** Kelley Newlin-Lew, MaryAnn Perez-Brescia, Veena Channamsetty, Mariana Salas-Vega, Maria Krol, Francky Jacque, Cathi Lippman, Nancy A Allen

**Affiliations:** 1 School of Nursing University of Connecticut Storrs, CT United States; 2 Community Health Center, Inc Middletown, CT United States; 3 Hispanic Alliance of Southeastern Connecticut New London, CT United States; 4 School of Nursing Southern Connecticut State University New Haven, CT United States; 5 Vascular Medicine Outcomes Program, Section of Cardiovascular Medicine Yale University New Haven, CT United States; 6 College of Nursing University of Utah Salt Lake City, UT United States

**Keywords:** community-based participatory research, continuous glucose monitoring, diabetes, federally qualified health center, familismo, feasibility, Hispanic adults, peer support, protocol, type 1 diabetes, mixed methods, participatory research

## Abstract

**Background:**

Hispanic adults with type 1 diabetes (T1D) have suboptimal access to continuous glucose monitoring (CGM). Widening access to and increasing uptake of CGM for Hispanic adults with T1D are warranted.

**Objective:**

This randomized controlled trial (RCT) will evaluate the feasibility of a federally qualified health center (FQHC) CGM intervention and assess for an intervention signal in patient outcomes.

**Methods:**

A mixed methods, pragmatic pilot RCT will be used. A total of 30 adult Hispanic patients with T1D will be recruited from 4 FQHC sites allocated to provide the intervention (n=2) or control (n=2) conditions. At intervention sites, participants must be willing to use CGM for 3 months and have a willing adult family member participate in the study. Guided by the socioecological model, our intervention has three levels: (1) individual (culturally sensitive CGM information, motivation, and skills acquisition), (2) family or social networks (integration of the core Hispanic values of *familismo* and *collectivismo* to leverage family and peer support for CGM uptake), and (3) health care provider levels with CGM training using Project ECHO (Extension for Community Healthcare Outcomes). Intervention participants (n=15) will receive a culturally sensitive CGM intervention with 4 weekly intervention sessions (coattended by a family member), followed by 7 peer support group sessions over 6 months. Control participants will receive a self-monitoring of blood glucose control condition over a 6-month period. Study feasibility will be assessed in terms of recruitment, enrollment, retention, adherence, study procedures and implementation, and acceptability with mixed methods. We will collect physiological (eg, glycated hemoglobin and CGM metrics) and psychosocial (eg, depression, quality of life, social support, and interpersonal processes of care) outcome data. Feasibility data will be analyzed using content analysis and univariate or bivariate statistics. Linear and generalized linear mixed modeling will assess intervention signals and clinically meaningful differences from baseline to 3 and 6 months.

**Results:**

Funding for this project was secured in September 2022. As of May 2024, recruitment commenced following formative qualitative data collection on the social determinants of health and CGM uptake in Hispanic adults with T1D (N=32). Our community advisory board informed protocol modifications by reviewing qualitative findings, collaborating on related intervention refinement, and advising on cultural sensitivity methods.

**Conclusions:**

Guided by the socioecological model, our novel FQHC CGM intervention will provide feasibility and outcome data to guide a full-scale RCT. Our intervention model has unique potential to widen CGM access and increase CGM uptake in low-income Hispanic adults with T1D while improving outcomes for this vulnerable population.

**Trial Registration:**

ClinicalTrials.gov NCT06487962; https://clinicaltrials.gov/study/NCT06487962

**International Registered Report Identifier (IRRID):**

PRR1-10.2196/60583

## Introduction

### Overview

Historically, minoritized people have higher risks for chronic health conditions, such as diabetes, and greater comorbidities that are concerning for patients, clinicians, and researchers. Several explanations have been suggested including socioeconomic factors, lack of quality of care, and barriers to access. One minoritized group, Hispanic adults with type 1 diabetes (T1D), has greater glucose instability with increased incidence of acute complications, both hypoglycemia and diabetes ketoacidosis, than non-Hispanic White people [1-3]. Hispanic adults with T1D thus have disproportionately higher rates of related emergency department (ED) visits, hospitalizations, and death [4-8]. Continuous glucose monitoring (CGM) may dramatically mitigate these health inequities.

CGM systems, such as the Abbott FreeStyle Libre, measure glucose levels manually and continuously. The Abbott FreeStyle Libre system includes an interstitial glucose sensor worn on the upper arm with the use of a reader or smartphone app. Manually, when the Libre 2 glucose sensor is scanned with the reader or smartphone app, visualization and storage of glucose values for interpretation are provided. The reader or smartphone app monitors glucose levels continuously, providing alarms for glucose thresholds of impending and actual hypoglycemia and hyperglycemia [9]. Compelling data reveal that both CGM use and an increased number of CGM scans are associated with improved clinical outcomes, such as reductions in glycated hemoglobin (HbA_1c_) and improved glucose stability (glucose time in range) with a significant decrease in the incidence of hypoglycemia and hyperglycemia [10-14]. Further, research shows that the use of CGM is related to a lower incidence of hospitalizations for acute diabetes complications, both diabetes ketoacidosis and severe hypoglycemia, among others [15-17].

Informed by rigorous research, CGM has emerged as an American Diabetes Association standard of care for diabetes management in adults on multiple daily injections or continuous insulin therapy [18]. Despite research and related recommendations for CGM, Hispanic adults have not equitably benefited from this technology. Hispanic adults with T1D, compared to their non-Hispanic White peers, have about a 2-fold lower rate of CGM use [19-21]. Moreover, there is a lack of intervention studies reported to increase CGM use in Hispanic adults with T1D. However, in one quality improvement study of non-Hispanic Black and Hispanic children and adults in 5 endocrinology centers, there was an increase in CGM use by 15% in Hispanic people with T1D [22]. In that quality improvement project, several approaches were used, including unconscious bias training, translation of educational materials into Spanish and other languages, social determinants of health (SDOH) screening and referrals, the use of CGM champions, standardized workflow for people with T1D on public or private insurance, and streamlining of communication among providers, durable medical equipment suppliers, and people with T1D. In research studies examining diabetes technology uptake in Hispanic adults with type 2 diabetes, several successful strategies have been identified. These strategies include cultural tailoring of interventions in Spanish and individualizing interventions to literacy levels, socioeconomic context, and cultural values and beliefs [23]. Other effective strategies include multimodal interventions [23], using peer mentors [24], involving family members [25], group education, and an in-person intervention component [23]. Thus, a multimodal approach, incorporating several of these intervention components, was used to design this study with an overarching goal of decreasing the disparity of CGM use in Hispanic adults with T1D cared for at federally qualified health centers (FQHCs).

### Theoretical Background

**Figure 1:**
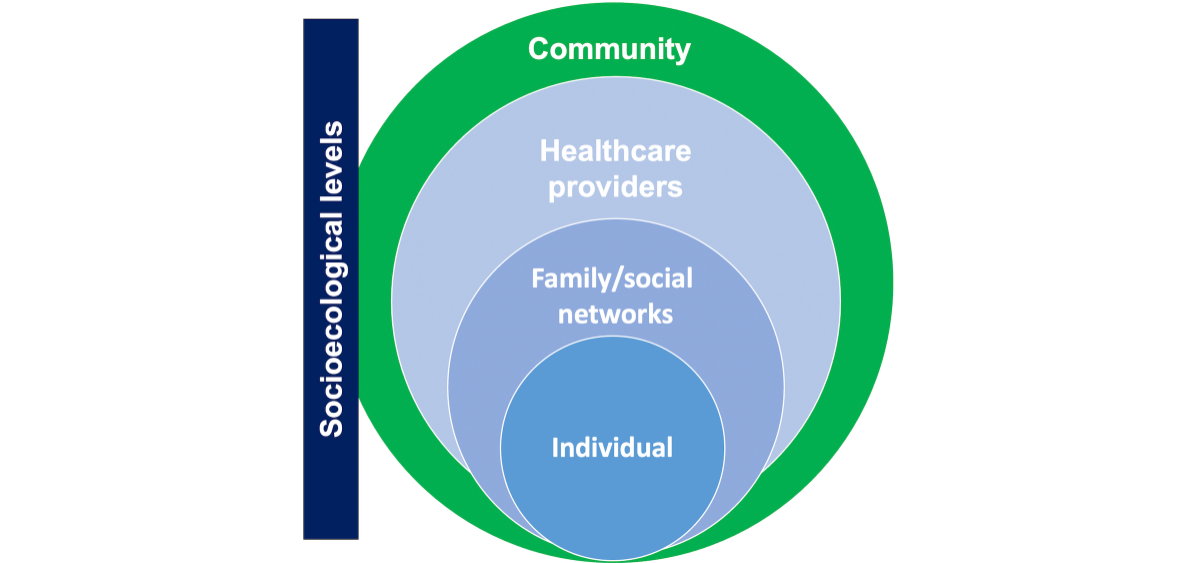
Socioecological model.

For Hispanic adults with diabetes, suboptimal CGM use is increasingly understood as a downstream effect of SDOH [26-28]. Framed by the socioecological model (SEM), SDOH may be conceived on multiple levels. In this study, the individual or Hispanic adult with T1D is nested by the community, health care provider, and family or social network levels (Figure 1). SEM goes beyond simply modifying health behaviors at the individual level by addressing the individual’s broader, multilayered context to affect T1D outcomes synergistically [29].

Most distal to the individual is the community level. In community-based participatory research, the community level may exert a favorable influence on the individual, ensuring that the health care provider and family or social network levels of the intervention are designed to meet the needs of the individual participants. Increasingly, community advisory boards (CABs) play a central role in ensuring that individuals or participants receive culturally relevant and competent care in clinical studies. CABs may serve as essential liaisons between the community and the research team, overseeing and participating in the research process [30-32].

Next, the health care provider level is concerned with access to quality T1D care. With respect to CGM use, the health care provider level has received important attention. Provider prescribing biases, both ethnic- and insurance-based biases, are well documented as contributing disparities in CGM use [33-35]. CGM access may be further curtailed by a national shortage of endocrine providers [36,37]. However, primary care providers (PCPs), particularly FQHC providers, are well poised to fill the gap in T1D care for Hispanic adults with bicultural and bilingual resources to meet patient needs [38]. Yet, PCPs report no or some confidence (70%) in using or interpreting CGM data with less aptitude in insulin titration than endocrinologists [39]. To overcome these barriers to quality T1D care, Project ECHO (Extension for Community Healthcare Outcomes) is a promising approach. Project ECHO is an established, interactive model to empower PCPs with subspecialty knowledge and training to improve health outcomes in primary care [40]. T1D-specific ECHO programs have been established to strengthen PCP abilities to manage T1D and widen access to CGM [41,42].

Proximally nesting the individual, the family or social network level concerns the social and community context. In terms of CGM use, the family or social network level remains understudied. However, the wider diabetes literature shows that family and community peer support may be critical for many Hispanic adults to successfully adopt diabetes self-management behaviors and, by extension, potentially CGM use [43-54]. Integrating family members in diabetes care and education leverages the Hispanic core value of *familismo*. *Familismo* emphasizes family support in diabetes self-management, with family advice primary in patient health care decisions [49,54-56]. Research further indicates that spousal support promotes patient empowerment in CGM use [55,57]. Community peer support taps the Hispanic cultural value of *collectivismo*—shared Hispanic ethnicity promotes self-disclosure and valuing of opinions in intracultural exchanges [58]. *Collectivismo* may facilitate rapport between patients and a peer supporter, with positive implications for health promotion and group learning reported [59-61]. Research reveals that Hispanic adults with diabetes are in favor of peer support, which has been found to promote CGM use [24,62].

**Figure 2:**
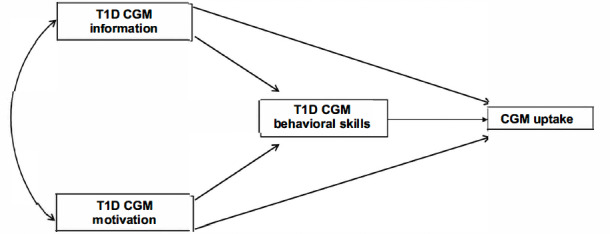
Modified IMB model for CGM uptake in Hispanic adults with T1D. CGM: continuous glucose monitoring; IMB: information-motivation-behavioral; T1D: type 1 diabetes.

The health care provider and family or social network levels exert considerable influence on the individual or Hispanic adult with T1D with respect to initiating CGM or not. To support initiation, culturally tailored behavioral approaches are warranted. In Hispanic adults with diabetes, the information-motivation-behavioral (IMB) skills model has been well validated [63]. Adapted for Hispanic adults with T1D, the IMB model posits that, while addressing motivations for behavior change, patient acquisition of CGM information or knowledge and skills collectively and respectively contribute to CGM use and adherence (Figure 2) [64].

### Study Purpose

The following study protocol details study methods to assess the feasibility and outcomes of our 3-level intervention, targeting Hispanic adults with T1D receiving care in an FQHC. Our 3-level intervention captures the socioecological levels of the health care provider (Project ECHO), family or social networks (integration of the core Hispanic values of *familismo* and *collectivismo* to leverage family and peer support for CGM uptake), and individual (culturally sensitive CGM information, motivation, and skills acquisition) levels. The pilot randomized controlled trial (RCT) intervention was refined by our highly engaged CAB. The CAB was formed by inviting Hispanic adults with T1D, their friends, and family members, and others concerned about T1D outcomes in Hispanic adults (eg, health care providers, leaders, and members of Hispanic community-based organizations, among others) were invited to join the CAB. The CAB used qualitative findings on SDOH and CGM uptake, collected from multiple stakeholder groups, to inform intervention refinement over 10 monthly 1-hour meetings. Vital to this process, the CAB reviewed additional study methods with advisement. The study will provide feasibility and outcome data on our FQHC intervention for CGM adoption and maintenance in low-income Hispanic adults with T1D.

### Study Aims

The aims of the study are as follows:

1. Assess study feasibility: (a) recruitment yield by type and retention (differential rates by participant characteristics), (b) retention, (c) implementation (data collection procedures, intervention fidelity, and dosages), and (d) acceptability.
2. Assess or estimate (a) significant intervention signals from baseline to 3 and 6 months (physiological and psychosocial outcomes), (b) relationship of dose to outcomes, and (c) clinically meaningful differences in T1D outcomes.

## Methods

### Study Design and Sampling

A mixed methods, pragmatic pilot RCT will be used to evaluate the study’s aims. Mixed methods will be used to assess study feasibility and acceptability, while quantitative methods will be used to assess RCT outcomes.

We randomized 4 Connecticut FQHC sites to serve as control (n=2) or intervention (n=2) sites. Inclusion and exclusion criteria are listed in Table 1. We plan to enroll 15 intervention and 15 control participants. Our sampling goal reflects that the RCT is primarily focused on the assessment of feasibility rather than intervention efficacy, as there is a lack of CGM intervention studies for Hispanic adults with T1D at FQHC. Therefore, the pilot study will test key aspects of the study design, such as recruitment strategies, data collection methods, intervention protocols, and participant engagement, before conducting a full-scale RCT. The total sample size of 30 will support the estimation of the proportions of participants who have “satisfactory” feasibility measures with SEs that cannot exceed ±0.1. Additionally, the “per group” sample sizes (15 a piece) will support the estimation of variances for outcome variables with sufficient precision to guide power analyses for a subsequent, full-scale RCT.

**Table 1:** Inclusion and exclusion criteria.

			Potentially eligible intervention participants		Potentially eligible control participants
**Inclusion criteria**					
	>18 years of age^a^	✓		✓	
	Hispanic^a^	✓		✓	
	Diagnosis of T1D^a,b^	✓		✓	
	FQHC^c^ PCP^a,d^	✓		✓	
	English- or Spanish-speaking^a^	✓		✓	
	Currently wearing or have you worn a continuous glucose monitoring device or continuous glucose monitor?	✓			
	If you are currently wearing or have you worn a continuous glucose monitor, are you struggling with this device?	✓			
	Adult family member or friend, who will give consent to participate in the study and coattend the 4-week intervention sessions	✓			
**Exclusion criteria**					
	Pregnant or planning to become pregnant	✓		✓	
	Lactating	✓		✓	
	Serious illness that may prevent study participation (eg, severe depression, advanced cancer, and advanced dementia)^a^	✓		✓	
	<6 months life expectancy^a^	✓		✓	
	Uncorrected hearing impairment^a^	✓		✓	
	Uncorrected vision impairment^a^	✓		✓	
	Alcohol or drug abuse or dependency^a^	✓		✓	

^a^Prescreening criteria.

^b^T1D: type 1 diabetes.

^c^FQHC: federally qualified health center.

^d^PCP: primary care provider.

### Recruitment

Potential control and intervention participants will be identified by the FQHC’s diabetes registry, specifically Hispanic adults with a T1D *ICD-10* (*International Statistical Classification of Diseases, Tenth Revision*) code. Potentially eligible control and intervention participants will be prescreened by FQHC project managers (Table 1). Upon passing the prescreening, 3 primary recruitment strategies will be operationalized. First, the FQHC’s patient engagement platform, Luma, will be used. The FQHC routinely uses Luma to text patients on their cell phones for reminders, such as upcoming appointments, among other purposes. Using the Luma platform, potential participants will be notified via 1-way text of the study with a link to its flyer (English or Spanish). FQHC project managers will send a study information letter, with an enclosed study flyer, to the potentially eligible patients with T1D. FQHC project managers will additionally call potential study participants to assess their interest in study participation. Those potential participants who show an interest in study participation will be asked to participate in a phone screening.

### Three-Level Intervention

The CGM intervention has three levels: (1) individual, (2) family or social networks, and (3) health care provider levels (Figure 1).

#### Individual Level

**Figure 3:**
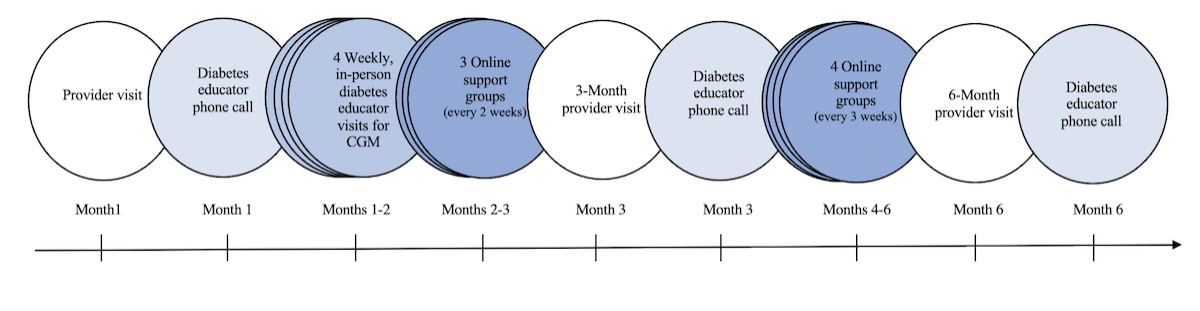
Intervention study timeline. CDCES: certified diabetes care and education specialist; CGM: continuous glucose monitoring.

At the individual level, the CGM intervention consists of 3 components. First, following the study’s 3 respective health care provider visits (see Health Care Provider Level section), intervention participants will be invited to participate in a post–provider visit phone call with the FQHC’s bilingual or bicultural Hispanic certified diabetes care and education specialist (CDCES) or registered nurse (Figure 3). During the postvisit phone call, the CDCES will review, explain, and answer questions regarding the provider’s T1D plan of care to ensure participant understanding and thereby promote related adherence.

Second, the CGM intervention includes 4 weekly, in-person, CDCES educational visits delivered by the study’s CDCES (Figure 3). The CDCES received 4 hours of training in preparation for delivering the educational visits. The CDCES visits will be held at the participant’s FQHC site of care. The visits are guided by the IMB skills model, adapted for CGM uptake [64]. The IMB-guided sessions provide culturally sensitive, interactive CGM sessions for each participant and their family member (referred to as a dyad) to promote information, motivation, and behavioral skills acquisition for effective uptake and behavioral maintenance of CGM (Figure 2). At week 2 of the CDCES educational sessions, the intervention participants will begin CGM, specifically the FreeStyle Libre 2 system (Table 2).

**Table 2:** Weekly content for certified diabetes care and education specialist (CDCES)–led continuous glucose monitoring (CGM) educational sessions.

Week	Session
1	Session 1 goals: Establish a therapeutic relationship with the dyad and introduce CGM. Confianza: Promote dyad trust with initiating and conducting sessions, attending to simpatia, personalismo, and respeto. Provide personalized education, attentive listening, and discussion. Information: Personalized approach in reviewing or introducing (1) the purpose of CGM and (2) glucose ranges with attention to low and high blood sugars. Use of bidirectional discussion, teach-back method, and culturally sensitive materials with pictorials and simple health messages. Motivation: Discuss health beliefs, motivating factors for CGM, concerns about CGM, and barriers to CGM use. Respect health beliefs; do not challenge them. If relationally appropriate and health belief is a barrier to CGM uptake, consider delicately reframing health belief. Skill development: Promotion of dyad familiarity with FreeStyle Libre 2 device (“Reader”), sensors, and sensor applicator through touching and holding. Encourage patient questions and bidirectional discussion with “hands-on” experience.
2	Session 2 goals: Foster a therapeutic relationship with the dyad and provide education (with goal setting) on hypoglycemia or hyperglycemia, related CGM use, and CGM sensor insertion. Confianza: See week 1. Information: See week 1. Additional content: Personalized approach to review or introduce (1) hypoglycemia and hyperglycemia and related treatment; (2) CGM trend arrows, glucose target ranges, and CGM alarms; and (3) CGM sensor insertion. Motivation: See week 1. Additional content: CGM goal setting with motivational interviewing. Discuss potential barriers that may impede the achievement of the goal. Collaboratively engage in preemptive problem-solving to generate potential solutions to overcome anticipated barriers to CGM use. Skill development: See week 1. Additional content: Following the registered nurse or CDCES insertion of the sensor, the participant practices in checking glucose with the sensor and understanding the Reader displays.
3	Session 3 goals: Foster a therapeutic relationship with the dyad and provide education on safely responding to CGM trend arrows or alarms and healthy dyadic communication. Confianza: See week 1. Information: See week 1. Additional content: Personalized approach to review or introduce (1) CGM trend arrows or alarms and safe responses, (2) concept of “insulin stacking” and effect on glucose levels, and (3) healthy dyad communication strategies to promote type 1 diabetes self-management. Motivation: See weeks 1 and 2. Skill development: See week 1. Additional content: Practice accessing and interpreting the Reader alarm screen. Use the teach-back method to enhance cognitive skills with respect to responding to alarms and turning off alarms. Dyad practice of healthy communication strategies.
4	Session 4 goals: Foster a therapeutic relationship with the dyad and provide a review of CGM and communication strategies, plus guide the participant in the independent insertion of the CGM sensor. Confianza: See week 1. Information: See week 1. Additional content: Personalized approach in (1) reviewing healthy communication strategies, (2) responding to CGM trend arrows or alarms and safely treating hypoglycemia and hyperglycemia, and (3) insertion of the CGM sensor. Motivation: See weeks 1 and 2. Skill development: See week 1. Additional content: Patient insertion of the CGM sensor using an applicator with guidance.

Each session will integrate *confianza* (trust), *respeto* (respect), motivation (health beliefs, motivating factors, and barriers), and skill development*.* The 4 weekly sessions further integrate the following components of discussion, “hands-on” learning, motivational interviewing, goal-setting, problem-solving, and bidirectional inquiry with an emphasis on a personalized approach and safety (Table 2). Educational handouts, adapted from the Share Plus Intervention, will be provided to the dyad to reinforce session content [65-68]. The educational materials use pictorials and low-literacy language. All educational materials have been reviewed by members of the target population with related modifications to ensure that the handouts are easily understandable and culturally appropriate. The teach-back method will be used at every session. This method provides a way of checking the understanding by asking participants to state in their own words what they need to know or do know.

Third, the CGM intervention includes a total of 7 virtual support group sessions (Figure 3). The virtual support group sessions will be held by a Hispanic bilingual, bicultural peer—an adult with T1D with >2 years of CGM use. The goal of the support group sessions is to reinforce information and skills gained during the 4-week CGM educational sessions and introduce new content concerning, for example, diet and physical activity (see Family or Social Network Level section). The virtual support group sessions will be held using the HIPAA (Health Insurance Portability and Accountability Act)-compliant Zoom (Zoom Video Communications) meeting platform.

#### Family or Social Network Level

At the family or social network level, the core Hispanic values of *familismo* and *collectivismo* will be leveraged to promote CGM uptake. Two components of the individual-level intervention will integrate (1) family members (or friends) and (2) a peer educator. A family member will coattend in-person CGM educational sessions (see Individual Level section) with each participant with T1D to provide support in acquiring CGM knowledge and skills and their application in real-world settings.

Previous studies reveal that education alone in Hispanic adults with diabetes is insufficient, and peer support may be warranted for successful behavior change [24,51,53,69-72]. Led by a Hispanic bilingual, bicultural peer with T1D, the virtual support group sessions will provide unique support [73]. Research suggests that the peer format will foster the Hispanic cultural value of *collectivismo* to promote participant self-disclosure and valuing of opinions, particularly as they pertain to CGM [58]. Moreover, the common bond created through the shared lived experience of T1D and initiating or using CGM, a bond not frequently shared between patients and their providers, will likewise promote support in CGM adoption. The Hispanic bilingual, bicultural peer received 10 hours of Diabetes Community Care Coordinator training from the Association of Diabetes Care and Education Specialists in preparation for delivering the virtual support group sessions [73].

#### Health Care Provider Level

Intervention providers (doctors of medicine [MDs], doctors of osteopathic medicine [DO], and advanced practice registered nurses [APRNs]) received intensive clinical support for T1D care in preparation for the study. We developed and deployed 2 Project ECHO programs to strengthen intervention provider knowledge and skills with a 4-week program in T1D insulin therapy and an 8-week program of Hispanic culturally sensitive CGM education and care (Table 3). Health care providers will see intervention participants at baseline, 3 months, and 6 months or more frequently as indicated (Figure 3).

**Table 3:** Project ECHO (Extension for Community Healthcare Outcomes) insulin and continuous glucose monitoring (CGM) content.

Week			Project ECHO topics
**Insulin therapy**			
	Week 1	T1D^a^ and review of insulins	
	Week 2	Selection of insulins-patient factors	
	Week 3	Dosing insulin	
	Week 4	Adjusting basal or bolus insulin doses	
**CGM education and care**			
	Week 1	CGM basics in Hispanic adults with T1D	
	Week 2	Using CGM to improve clinical outcomes in Hispanic adults with T1D	
	Week 3	Accessing CGM data	
	Week 4	Implementation of CGM in Hispanic adults with T1D	
	Week 5	CGM payor coverage and reimbursement	
	Week 6	Barriers and strategies to optimize CGM use in Hispanic adults with T1D	
	Week 7	Counseling Hispanic adults with T1D on using CGM	
	Week 8	Case studies	

^a^T1D: type 1 diabetes.

### Control Condition

**Figure 4:**
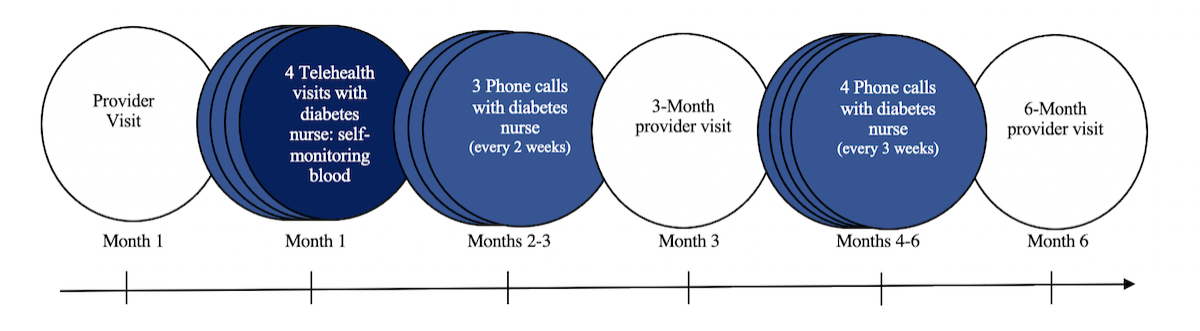
Control study timeline. CDCES: certified diabetes care and education specialist; SMBG: self-monitoring of blood glucose.

The control condition consists of three components: (1) virtual telehealth visits, (2) support phone calls, and (3) self-monitoring of blood glucose (SMBG)–informed T1D insulin therapy (Figure 4).

#### Virtual Telehealth Visits and Support Phone Calls

Four virtual telehealth visits will be delivered via the HIPAA-compliant Zoom meeting platform by a bilingual (English or Spanish) Hispanic CDCES (Figure 4). Session content focuses on increasing participant knowledge and skills related to SMBG. SMBG topics include T1D and blood glucose levels; blood glucose targets, measurement, and monitoring; and causes and management of hypoglycemia and hyperglycemia. American Diabetes Association and Centers for Disease Control educational materials, available in Spanish or English, will be used to guide each session. In addition, control participants will have 5 phone calls with the Hispanic CDCES (Figure 4). The goal of these phone calls is to reinforce SMBG testing knowledge and skills.

#### T1D Insulin Therapy

Control providers (MDs, DOs, and APRNs) participated in an in-depth, 4-week ECHO program on T1D insulin therapy (Table 3). Control site providers will manage insulin therapy for participants informed by respective HbA_1c_ levels and SMBG data. Control site providers will see participants at baseline, 3 months, and 6 months or more frequently if clinically warranted (Figure 4).

### Measures and Data Management

Data will be collected at baseline, weekly, 3 months, and 6 months. Data will be entered into a REDCap (Research Electronic Data Capture; Vanderbilt University) [74,75] database by trained data collectors who are fluent in English and Spanish (Table 4 and Textbox 1).

**Table 4:** Background and outcome measures.

			Baseline		Weekly		3 months		6 months
**Demographics**									
	Age	✓							
	Income	✓							
	Education	✓							
	Gender	✓							
	Language preference	✓							
	Nativity	✓							
	Time living in the United States	✓							
**Health-related factors**									
	Year of T1D^a^ diagnosis	✓							
	Primary insurance	✓							
	Chronic diabetes complications	✓							
	Number of hypoglycemic-related ED^b^ visits over the past 3 months	✓				✓		✓	
	Number of hypoglycemic-related hospitalizations over the past 3 months	✓				✓		✓	
	Number of hyperglycemic-related ED visits over the past 3 months	✓				✓		✓	
	Number of hyperglycemic-related hospitalizations over the past 3 months	✓				✓		✓	
**Glucose control**									
	HbA_1c_^c^	✓				✓		✓	
**Depression**									
	Patient Health Questionnaire	✓				✓		✓	
**Quality of life**							✓		✓
	Medical Outcomes Survey Short Form-36	✓				✓		✓	
**Social support**									
	Perceived Social Support from Family Scale	✓				✓		✓	
**Patient-provider trust**									
	Interpersonal Processes of Care Survey: Short-Form	✓				✓		✓	
**CGM^d^ metrics (intervention participants only)**									
	Glucose time in range					✓		✓	
	Glucose time below range					✓		✓	
	Glucose time above range					✓		✓	
**SDOH^e^ factors**									
	PRAPARE^f^	✓							
	Transportation to 4 weekly CDCES^g^ in-person visits (study month 1)			✓					

^a^T1D: type 1 diabetes.

^b^ED: emergency department.

^c^HbA_1c_: glycated hemoglobin.

^d^CGM: continuous glucose monitoring.

^e^SDOH: social determinants of health.

^f^PRAPARE: Protocol for Responding to & Assessing Patients’ Assets, Risks & Experiences.

^g^CDCES: certified diabetes care and education specialist.

**Selected PRAPARE (Protocol for Responding to & Assessing Patients’ Assets, Risks & Experiences) items.**

At any point in the past 2 years, has season or migrant farm work been your or your family’s main source of income?

- Nominal level of measurement

Have you been discharged from the armed forces of the United States?

- Nominal level of measurement

What is your housing situation today?

- Nominal level of measurement

Are you worried about losing your housing?

- Nominal level of measurement

What is your current work situation?

- Ordinal level of measurement

In the past year, have you or any family members you live with been unable to get any of the following when it was really needed? (Food and utilities, clothing, childcare, medicine or any health care, and phone)

- Nominal level of measurement, respectively

Has a lack of transportation kept you from medical appointments, meetings, work, or getting things needed for daily living?

- Nominal level of measurement

How often do you see or talk to people who you care about and feel close to? (For example, talking to friends on the phone, visiting friends or family, going to church, or club meetings)

- Ordinal level of measurement

Are you a refugee?

- Nominal level of measurement

#### Background Factors

Demographics, health-related factors, and SDOH will be measured (Table 4). At baseline, demographic (eg, nativity, race, ethnicity, and language preference) and health-related (ie, duration of T1D and specific diabetes chronic complications) data will be collected. SDOH will be measured at baseline and weekly. At baseline, SDOH will be measured with PRAPARE (Protocol for Responding to & Assessing Patients’ Assets, Risks & Experiences). The nationally standardized PRAPARE, developed by the National Association of Community Health Centers, is routinely used at the study FQHC to support social needs screening in its primary care settings. PRAPARE is a 21-item, unsummed measure available in English and Spanish. It will be administered at baseline with data capture of selected items (items 3-8, 11, 14-16, 19; Textbox 1) [76]. Upon identifying a social risk, the CDCES, as indicated, will refer participants, for example, to 211 Connecticut. Accessible by phone or online, 211 Connecticut provides referrals to a wide range of health and human service resources, including transportation, basic needs, housing, and food, among several others. Additional SDOH data (eg, participant transportation to study visits) will be collected weekly for each of the CDCES educational sessions (Textbox 1).

#### Primary Outcome: Feasibility

In this study, feasibility will be assessed in terms of (1) recruitment and enrollment, (2) retention, (3) adherence, (4) study procedures and implementation, and (5) acceptability (Textbox 2).

**Feasibility and acceptability measures.**

**Recruitment and enrollment**

- Number screened per month
- Number enrolled per month
- Proportion of eligible screens enrolled
- Reason for study refusal
- Recruitment yield by type

**Retention**

- Retention rate by intervention or control group
- Retention differential rates by participant characteristics
- Proportion of completed data collection points

**Adherence**

- Continuous glucose monitoring use (number of days worn, % time active)
- Intervention and control schedule (% followed)

**Study procedures or implementation**

- Data collection fidelity
- Certified diabetes care and education specialist (CDCES) intervention fidelity
- Peer-led support group fidelity
- Intervention and control doses

**Acceptability**

- Intervention acceptability (CDCES, peer, and providers, respectively) by focus group interviews

During screening visits, demographic characteristics will be entered into our REDCap recruitment and enrollment database. The recruitment and enrollment database will track recruitment sources, eligibility screening, and enrollment outcomes. Qualitative data on the rationale for study refusal will also be captured in the database. The database will be used to inform modifications to our recruitment strategies to optimize our yield of enrolled participants. In addition, the recruitment and enrollment database will be linked to a retention database. The retention database will track retention yields, retention by differential rates by participant characteristics, and the proportion of completed data by groups (intervention or control).

The REDCap database will be used to track adherence to the intervention and control schedules (Figures 3 and 4). For intervention participants, data on the number of days the CGM was worn and the percentage of time the CGM was active will be collected from the electronic health record (EHR), specifically an electronic copy of the CGM report after each provider visit at 3 and 6 months.

Using the REDCap database, participant intervention and control doses will also be tracked. The number of provider visits completed (3 planned study visits and additional visits as indicated) will be tracked over the 6-month study period. For all participants, the completed number and duration of CDCES and peer-led intervention or control components will be recorded.

Addressing the fidelity of study procedures, data collectors received 6 hours of training in using REDCap in preparation for the RCT. Following training, direct observation revealed that data collectors had achieved high competency in related skills acquisition. During the RCT, fidelity of data collection will be assessed by self-report. Prior to completing a REDCap module for a participant, the database requires that related data entry be endorsed as verified, completed, or incomplete. In addition, in REDCap, open-ended data will be entered on any problems encountered with data collection at baseline, 3 months, and 6 months for each study participant.

With respect to intervention fidelity, specifically the 4 weekly CGM educational sessions, we will use self-report and in vivo observation assessments. A fidelity checklist was developed and integrated into the FQHC’s EHR for each of the 4 sessions, respectively. Using the EHR, the CDCES will have a dedicated template for each of the 4 sessions, corresponding with the goals and content for each session, as displayed in Table 2. The templates have structured domains for entry of quantitative data (achievement of specific session goals and delivery of planned content or not) and corresponding qualitative data, such as participant motivation and goals for CGM, among others. The CDCES will self-report achievement of specific session goals and delivery of planned content or not by using a parallel checklist embedded in each of the session templates. Further, the CDCES will be randomly observed for adherence and competence in delivering the intervention protocol using a fidelity measure designed for this study.

Fidelity of the virtual support group sessions will be assessed by self-report using a web-based data fidelity system. For each of the 7 support group sessions, respectively, the web-based system will capture quantitative and qualitative data. The peer educator, for each session, will be prompted to endorse yes or no regarding the achievement of session goals and delivery of planned content in addition to entering parallel open-ended data.

Acceptability will be assessed with focus group interviews (FGIs) to be held with intervention participants, providers, and CDCES, respectively. FGI interview guides will pose questions on aspects of study processes or procedures (eg, recruitment, retention, data collection, intervention content, frequency of contact, duration of contact, and interventionist delivery).

#### Secondary Outcomes

Physiological outcomes are HbA_1c_, time in range, time below range, time above range, and frequency of hypoglycemic- or hyperglycemic-related ED visits or hospitalizations (Textbox 1). HbA_1c_ will be measured with the DCA 2000 point-of-care analyzer with a capillary blood sample, the standard measure of HbA_1c_ determination at the study FQHC. CGM glycemic metrics will be captured from FreeStyle Libre View reports (intervention participants only). The frequency of hypoglycemic- or hyperglycemic-related ED visits or hospitalizations over the past 3 months will be based on participant self-report (Textbox 1).

Psychosocial outcomes are depression, quality of life (QOL), social support, and interpersonal processes of care. All psychosocial measures are available in English and Spanish. Depression will be assessed with the 2-item Patient Health Questionnaire (PHQ) and 9-item PHQ. The PHQ-2 is a screening tool for depression. The PHQ-2, using the first 2 items of the PHQ-9, inquires about depressed mood and anhedonia over the past 2 weeks with item scores ranging from 0=not at all to 3=nearly every day. A total PHQ-2 score of >3 suggests major depressive disorder and warrants administration of the PHQ-9 [77]. PHQ-9 item scores likewise range from 0 to 3 (maximum score of 27). A threshold score of >10 indicates mild major depression, >15 or higher indicates moderate major depression, and >20 indicates severe major depression [78].

QOL will be measured by the Medical Outcomes Survey Short Form-36. This well-validated and reliable measure has 8 sections to capture distinct domains of QOL. Each section yields a scored scale, which is a weighted sum of the items in each section. Pooled scores are calculated to generate a total score, ranging from 0 to 100, with lower and higher scores indicating less or greater health-related QOL, respectively (Textbox 1) [77-79].

Social support will be assessed with the Multidimensional Scale of Perceived Social Support (MSPSS). The 12-item MSPSS assesses the perceived adequacy of support from family, friends, and significant others with corresponding subscales. The MSPSS is presented in a 7-point Likert-type format. Response options range from 1=very strongly disagree to 7=very strongly agree, with higher scores revealing greater levels of perceived social support. MSPSS subscale and total scale scores are computed with a specified formula. The MSPSS has demonstrated validity and reliability in Hispanic populations [80,81].

The Interpersonal Processes of Care Survey Short Form measures doctor communication, decision-making, and interpersonal style over the past 12 months. The 18-item Short Form was modified for this study to assess provider (MD, DO, and APRN) communication over the past 3 months. The Interpersonal Processes of Care Survey Short Form has 7 subscales presented in a 5-point Likert-type format; response choices range from 1=never to 7=always. The 3 communication subscales measure lack of clarity (2 items), elicit concerns and responses (3 items), and explain results (2 items). The decision-making subscale assesses patient-centered decision-making (1 item), while 3 interpersonal style subscales measure emotional support and compassion (3 items), discrimination due to race or ethnicity (2 items), and disrespectful office staff (3 items). For each subscale, item responses are averaged with a range of 1-5, with higher scores indicating greater levels [82].

### Ethical Considerations

The study procedures were approved by the Biomedical Research Alliance of New York Internal Review Board (Pilot RCT: FQHC Intervention for Uptake of CGM in Hispanic Adults With T1D, 24-08-011-910). Consistent with best practices in research, informed consent will be collected from the person with diabetes and the family member, and they can opt out at any time. All data will be deidentified to protect the privacy of participants. Intervention participants will be compensated following each study activity with a total amount of US $100 at baseline and up to US $900 (intervention participants with T1D) or US $500 (friend or family member of intervention participants with T1D) at 3 months after baseline. Intervention participants with T1D will be compensated up to US $700 at 6 months after baseline. In addition, control participants will be compensated following each study activity with a total amount of US $100 at baseline and up to US $550 at 3 months after baseline and US $600 at 6 months after baseline.

### Data Analytic Plan

#### Study Aim 1

Three aspects of study feasibility will be evaluated using a combination of univariate and bivariate statistical methods. Recruitment sources along with rates and levels of participant retention will be quantified relative to sites and time. Qualitative data on the rationale for study refusal, with the use of data coding, will be quantified. Participant characteristics (eg, age, gender, country of origin, and time since T1D diagnosis) will be summarized using means and SDs or frequencies and percentages, as appropriate to the level of measurement. Associations between participant characteristics and dropout frequency will be investigated via cross-tabulations. Retention will be assessed by participant adherence to the study schedule (Figures 3 and 4). Rates and patterns of adherence to intervention and control procedures and attendance throughout the study duration will be described. Acceptability of the intervention among FQHC providers, CDCES, and intervention participants will be appraised through content analysis of posttrial FGIs [83], using Graneheim and Lundman [84] qualitative content analysis approach to increase trustworthiness. This coding approach involves reading the FGIs several times and identifying and condensing meaning units that are words, sentences, or paragraphs containing aspects related to each other through their content and context. Next, the condensed text will be abstracted with codes, categories, and themes to best represent the perspectives of FQHC providers, CDCES, and intervention participants, respectively.

Completeness and validity of data collection procedures will be assessed by documenting the frequency of missing data and by checking for inconsistent or out-of-range values for all study variables. In-text data on barriers to data collection will be analyzed qualitatively using content analysis [83]. Successful implementation of study procedures will be evaluated via debriefing of study staff and documentation of any departures from the study protocol.

#### Study Aim 2

To quantify intervention signals, we will begin by contrasting HbA_1c_ levels between the intervention and control participants. To do this, we will apply linear mixed modeling (LMM) using study arm assignment (intervention vs control) and time (3 and 6 months) as “main effects,” the corresponding baseline value of HbA_1c_ as a covariate, and FQHC site variable as well as the “participant” identifier as random effects. This approach will support statistical testing of intervention effects on the mean value of HbA_1c_ while controlling for differences in baseline levels of the variable, accounting for clustering effects with study sites, and adjusting for correlations within participants over time. After using this strategy to assess the main effect of the intervention on the mean value of HbA_1c_, we will investigate the possibility of “intervention-by-time” and “intervention-by-site” interactions and the possibility that any imbalances in baseline participant characteristics (sociodemographic variables and health history factors) between study groups might confound valid estimation of intervention effects. Because the RCT is primarily concerned with feasibility assessment and will involve small samples with limited statistical power, the focus of these analyses will be “effect estimation” rather than formal statistical testing. In the second stage of analysis, we will apply the same modeling strategy to contrast the other physiological outcomes and the psychosocial measures between the intervention and control participants.

Throughout our analyses using LMM, we will attempt to respond to evidence of nonnormality in any of the outcome measures by applying normalizing transformations. If no transformation achieves approximate normality (as is likely to be the case for the time in range, below range, and above range variables as well as the counts of hypo- and hyperglycemic clinical events), we will, instead, apply a generalized linear mixed model with a link function appropriate to the distribution of the outcome measure. Mixed models of repeated measures are robust to the effects of missing observations that occur “at random.” Depending on the extent of missing data (while acknowledging restrictions imposed by the study’s small sample size), sensitivity analyses based on multiple imputations may be conducted to evaluate the potential impact of nonrandom missing data on estimates of the intervention effects.

For members of the intervention arm of the RCT, participant “dose” of the intervention will be described with a combination of means or SDs and frequencies or percentages. Additional analyses using LMM methods will be conducted to evaluate whether measures of intervention dose are associated with differential mean levels of the physiological and psychosocial outcome variables at the 3- and 6-month time points. Finally, we use results of LMM analyses for the following: (1) to calculate standardized effect sizes (Cohen *d* and eta-squared) for the intervention versus control condition on each of the physiological and psychosocial outcome measures, (2) to determine variance estimates for these outcome variables, (3) to quantify clustering effects (in the form of intraclass correlation coefficients) introduced by the study sites, and (4) to document temporal correlations of outcome measures within participants across the 3 study time points (baseline, 3 months, and 6 months). These statistics will be helpful, but not fully definitive (due to the small sample size), in guiding design decisions and statistical power analyses in preparation for a future, full-scale RCT of the CGM intervention.

## Results

Funding for this project was secured in September 2022. Formative qualitative data collection followed to examine SDOH and CGM uptake in Hispanic adults with T1D (N=32) to tailor the intervention to the target population. Our CAB informed modifications to the study protocol by reviewing and interpreting the qualitative findings, collaborating in the related refinement of the intervention, and advising on additional study methods and their cultural sensitivity. With institutional review board approval secured (February 2024) for the CAB-informed protocol, the first study participant was enrolled in May 2024. The main findings from the study will be presented at conferences and reported in peer-reviewed publications in collaboration with our CAB.

## Discussion

### Principal Findings

Guided by SEM, the expected outcome of our novel FQHC CGM intervention is the demonstration of pragmatic trial feasibility in terms of recruitment and enrollment, retention, protocol adherence, acceptability, and the development and implementation of the study procedures. A secondary expected outcome is that there will be a clinically significant intervention signal in HbA_1c_ between the groups. Additionally, we will obtain an estimation of variances for outcomes to guide power analyses and follow-up for a fully powered RCT. Our CGM intervention model, incorporating the SDOH, has the unique potential to widen access to and increase CGM use and acceptance in low-income Hispanic adults with T1D while improving outcomes for this vulnerable population.

In 2023, the Treatment and Complications Subcommittee of the National Clinical Care Commission provided a report to Congress on leveraging federal policies and programs to improve diabetes treatment and reduce complications [85]. This report supports the strategies used in this study. At the practice level, it was identified that there is a need for programs that support team-based care and develop the capacity to support technology-enabled mentoring interventions for people with diabetes. Additionally, the report identified a need to train the health care workforce to meet these practice needs. This report highlights the need for multicomponent diabetes technology interventions that focus on the SEM’s components, which is this study’s guiding theoretical framework.

This study will provide feasibility data to address the gap in CGM interventions designed to address the needs of Hispanic adults with T1D. A recent qualitative study of CGM acceptance in participants with type 2 diabetes cared for at FQHCs supports several of the approaches used in this study [86]. In that study, 6 themes were found that increased an individual’s self-efficacy for using CGM, which included initial expectations and overcoming initiation barriers, convenience and ease promoting daily use, increased knowledge leading to improved self-management, collaboration with provider and clinical team, improved self-reported outcomes, and barriers and burdens that are generally tolerated. Although this was a study in individuals with type 2 diabetes, these findings support several intervention components used in this study and the study design using the IMB model for behavior change.

There are several strengths of this study. First, the intervention development was guided by the SEM and included three levels: (1) the individual (culturally sensitive CGM information, motivation, and skills acquisition), (2) family or social networks (integration of the core Hispanic values of *familismo* and *collectivismo* to leverage family and peer support for CGM uptake), and (3) health care provider levels with CGM training using Project ECHO. Second, the intervention and study protocols were informed by a CAB that provided crucial insights into an individual’s and family’s needs, priorities, and perspectives that increased the cultural relevance, accessibility, and likelihood of adoption. Finally, as part of this study, we developed and deployed 2 Project ECHO programs to strengthen intervention provider knowledge and skills with a 4-week program in T1D insulin therapy and an 8-week program of Hispanic culturally sensitive CGM education and care. These strengths increase the likelihood of success of this pilot study.

A limitation of the proposed study is the small sample size for the RCT. However, the benefits of conducting a small pilot study include identifying potential issues early and providing the opportunity to refine the study design and methodology, improving participant recruitment strategies, assessing feasibility, optimizing data collection methods, and thus, increasing the likelihood of a subsequent RCT. Additionally, the study results may not be generalizable to other health care settings outside of FQHCs, such as clinics seeing people with private insurance and higher incomes. However, there is a great need to increase access to and use of CGM among patients cared for at FQHCs, especially for historically minoritized populations such as Hispanic adults with T1D.

This study was designed to overcome many potential barriers to CGM adoption in Hispanic adults with T1D cared for at FQHCs. For example, Project ECHO was incorporated into the research design at the health care level to educate FQHC providers and clinicians. One Project ECHO session was dedicated to the barriers and strategies to optimize CGM use in Hispanic adults with T1D. At the individual level, the intervention sessions were designed to address health beliefs, motivation factors for CGM, and concerns and barriers to CGM use, as well as CGM skill development. The intervention design included motivational interviewing strategies to allow for a collaborative engagement in preemptive problem-solving and to generate potential solutions to overcome anticipated barriers to CGM use. Moreover, several strategies to increase the feasibility of the pilot study were incorporated into the study design. These include having a Hispanic CAB, a bilingual-bicultural interventionist, bilingual-bicultural program managers responsible for recruitment, and a research team with expertise in conducting research in the Hispanic community.

### Conclusions

This study was designed to create a multicomponent novel intervention to increase CGM use in Hispanic adults with T1D cared for at an FQHC. The overarching aim of this pilot study is to determine intervention feasibility that will inform a future, larger R01-funded RCT of this FQHC-CGM intervention.

## Abbreviations

APRN: advanced practice registered nurse
CAB: community advisory board
CDCES: certified diabetes care and education specialist
CGM: continuous glucose monitoring
DO: doctor of osteopathic medicine
ECHO: Extension for Community Healthcare Outcomes
ED: emergency department
EHR: electronic health record
FGI: focus group interview
FQHC: federally qualified health center
HbA1c: glycated hemoglobin
HIPAA: Health Insurance Portability and Accountability Act
ICD-10: International Statistical Classification of Diseases, Tenth Revision
IMB: information-motivation-behavioral
LMM: linear mixed modeling
MD: doctor of medicine
MSPSS: Multidimensional Scale of Perceived Social Support
PCP: primary care provider
PHQ: Patient Health Questionnaire
PRAPARE: Protocol for Responding to & Assessing Patients’ Assets, Risks & Experiences
QOL: quality of life
RCT: randomized controlled trial
REDCap: Research Electronic Data Capture
SDOH: social determinants of health
SEM: socioecological model
SMBG: self-monitoring of blood glucose
T1D: type 1 diabetes

## Data Availability

The datasets generated or analyzed during this study are available from the corresponding author upon reasonable request.
